# Experimental Investigation on the Effects of Tool Geometry and Cutting Conditions on Machining Behavior during Edge Finishing of Granite Using Concave and Chamfered Profiling Tools

**DOI:** 10.3390/mi15030315

**Published:** 2024-02-24

**Authors:** Wael Mateur, Victor Songmene, Jules Kouam

**Affiliations:** Department of Mechanical Engineering, École de Technologie Supérieure (ÉTS), Montreal, QC H3C 1K3, Canada; wael.mateur.1@ens.etsmtl.ca (W.M.); jules.kouam@etsmtl.ca (J.K.)

**Keywords:** granite, edge finishing, cutting conditions, tool geometry, cutting forces, surface finish

## Abstract

Granite edge finishing through grinding is a common process in the granite processing industry, crucial for achieving the final desired shape and edge quality of products. This study focuses on the granite industry, specifically delving into the significance of grinding and polishing for improving aesthetics and extending material longevity. The experimental design entails a comprehensive factorial experiment plan involving two workpiece materials (white and black granite samples) and two cutting tool edge shapes (chamfer and concave), each with two grit sizes: G150 and G600. The cutting conditions varied and consisted of variations in spindle speeds (1500, 2500, 3500 rpm), feed rates (500, 1000, 1500 mm/min), and lubrication modes (wet/dry). The results uncover intricate relationships among these parameters and part quality, underscoring the pivotal role of tool geometry in achieving superior surface finishes and in controlling the cutting forces. These findings contribute to a nuanced understanding of the dynamic interplay between tool characteristics, material properties, and machining conditions within the granite industry.

## 1. Introduction

Polishing stands as a pivotal facet in the granite and marble processing industries, holding the key to not just enhancing aesthetics but also prolonging the life of materials. Granite plays a pivotal role in Quebec, Canada, influencing both its economy and culture. The granite industry significantly contributes to the provincial economy by creating jobs and fostering growth, establishing Quebec as a key global exporter of granite products. Beyond its economic impact, Quebec’s granite is crucial to the province’s architectural heritage, prominently used in building construction. The enduring and aesthetically valuable qualities of this granite hold cultural significance, representing the region’s history and identity. As a natural resource, Quebec’s granite not only signifies economic prosperity but also underscores the importance of preserving cultural heritage and responsibly managing the province’s geological wealth [1]. These granite products are used in kitchens, landscaping, and urban design. They are transformed using various processes, among which are sawing, milling, drilling, grinding, and polishing on the surface and edges. For many applications, the surface finish is the most required criterion. The obtained surface finish will depend on the granite material mineral composition but also the abrasive grit sizes, cutting process parameters, and conditions.

According to Sanmartin et al. (2011) [2], the selection of ornamental granites for new constructions is primarily based on their aesthetic properties because these define the architectural harmony of the construction with the surroundings and enhance the visual perception of the building. Surface finishes differ in surface roughness and affect slab aesthetics because they induce variations in the perceived texture (visual characteristic and tactile quality of the surface of a material), color, and gloss. Sanmartin et al. (2011) [2] found that roughness induced by different types of surface finish on the granite surface causes a change in the color (in most cases perceptible), primarily because of changes in the lightness parameter’s values, and that an inverse relationship exists between gloss and roughness only for low values of roughness. According to Sousa et al. (2013) [3], the systematic evaluation of the color, roughness, and gloss properties of the surface during rock processing enables the determination of the natural variation of the rocks and facilitates the distinction of the influence arising from processing-related variations.

López et al. (2018) [4] investigated the influence of the commercial finishes (polished, honed, disc cutting, and bush hammering) of ornamental granites on roughness, color, and reflectance. They found direct correlations between roughness average (Ra) and lightness on the one hand and between roughness and reflectance; the rougher the surface, the higher the lightness and reflectance values will be. They also found that the differences observed in roughness values and intensity of reflectance spectra obtained for each finish and granite tested could best be explained by the granite’s grain size and quartz content. Huang et al. (2002) [5] studied the surface finish and gloss of granites machined by grinding, finding that surface gloss improved as the grinding process progressed towards finishing, and that the dominant material removal mechanism varied from brittle (rough) to ductile (finishing), i.e., with decreasing abrasive grain size. They also found that surface roughness decreased with increasing ductile flow over the surface, while gloss was directly attributed to surface roughness. High gloss corresponds to low roughness, and the latter is formed during grinding in ductile mode.

The effect of the ductile material removal mode associated with the use of finishing abrasives was also pointed out by Saidi et al. (2015) [6] and was associated with high pressures, high cutting forces, and low coefficient of friction during surface polishing of granites. At an early stage of the polishing process, when using coarse abrasives with grit sizes under 400, the material removal mechanism was brittle fracture, detected by the increase in the friction coefficient and the decrease in the contact pressure. At the end of the polishing process, the material removal mechanism was ductile flow. Xu et al. (2003) [7] have studied the material removal mechanisms in sawing and diamond grinding of granite. They found that ductile flow prevails during grinding, especially for grinding with finer grits, and that the different removal mechanisms were closely related to the normal grinding force, which is governed by the grinding depth. During the sawing, the mineral composition of the granite affected the material removal mechanisms; biotite revealed more fracture modes as compared to feldspar and quartz. The authors recommended careful selection of machining parameters and tools in order to minimize ductile flow in sawing and maximize it during fine grinding. Huang and Xu (2003a) [8] revealed that the formation mechanism of gloss on a ground granite surface is mainly mechanical rather than chemical and that gloss readings increased when the Ra values of the surface decreased following an exponential relationship. They also added that the surface roughness depends on the amount of ductile flow on the ground surface. Huang and Xu (2003b) [9] found that the feed rates did not affect the gloss readings when grinding granites; the gloss readings appeared to be more dependent on tool rotational speed and tool workpiece contact pressure; thus, this is related to the workpiece material’s composition and hardness. According to Tanovic et al. (2011) [10], granite machining involves both brittle fracture and plastic deformation. Fragile fracture indicates that there are two mechanisms of crack propagation: medial cracks and lateral/radial cracks to chip formation. When micro-cutting granite, the initial ductile flow gradually transforms into brittle fracture once the critical cutting depth is reached.

Songmene et al. (2018) [11] observed that polishing conditions (feed rate, spindle speed) and tool path strategy can be used to reduce dust emission to some extent while maintaining the part quality and productivity at acceptable levels during granite polishing. The use of higher feed rates and higher polishing speeds led to better granite part surface finishes. Sun et al. (2022) [12] investigated the impact of machining parameters on the mechanism of surface formation and chip features of granites and found that the machining parameters have a stronger influence on machined granite surface quality, crack formation, and chip size. Especially, for the granite material removal mechanism, the strain rate was indicated as being the main determinant factor; higher strain rates (higher than 5 × 10^4^/s) produced better surface quality and smaller chips.

Some machining research works were also carried out on composite materials containing granite particles. Ghorbani et al. (2018) [13] studied the drilling of epoxy granite using both coated and uncoated carbide spiral drill bits. Their findings revealed that an optimal surface roughness (Rz) is attained at a low spindle speed and a high axial feed rate. Conversely, Ramesh et al. (2022) [14] reported a contrasting observation when drilling glass fiber/nano granite particle-reinforced epoxy composites. They found that increasing the speed and decreasing the feed rate resulted in a reduction of surface roughness. Adding another perspective, Quadros et al. (2014) [15] conducted a study highlighting the statistical analysis of the drilling performance of granite fiber-reinforced epoxy composites; they found that the feed rate has the most substantial influence on both thrust and torque behavior during the drilling process, ranging from approximately 52% to 68%, while the impact of cutting speed is comparatively lower, varying from approximately 15% to 35%. Overall, these varied findings underscore the complexity of the relationship between process parameters and part quality in composite material machining, emphasizing the need for a nuanced approach depending on the specific composition and characteristics of the materials involved.

Centering specifically on granite as the stone material, Songmene et al. 2018 [11] summarized research studies dealing with the effects of polishing parameters and tool paths during rotational plane polishing on part quality and dust emission; the authors observed that heightened speeds, increased feed rates, and larger abrasive grit sizes collectively yielded superior surface finishes. Furthermore, their exploration into tool paths revealed that the spiral trajectory enhanced part surface finish, while the linear path resulted in surfaces with higher Ra values. The surface finish and the energy required for granite shaping are also affected by the cutting fluid used. Rao and Nelson (2014) [16] analyzed the polishing of granite tiles with water and with water plus oil, using grit sizes ranging from 36 to 800. They concluded that, for all stages of polishing, i.e., for all grit sizes tested, the power consumption was higher when using a cutting fluid consisting of water plus oil as compared to the use of water only. The increase in power consumption ranged from 11% to 60%.

Most of the above-analyzed research works were carried out on plane surface finishing using single and multiple abrasives; very little research has been carried out on edge finishing processes. Also, a wide variety of profiling tools are available on the market for the edge finishing of natural and artificial stones, but there is very limited research work on their performance in wet or dry cutting conditions. Zhang et al. (2013) [17] investigated the cutting force during the machining of the irregular surfaces of granite using a diamond profiling wheel. They found that the larger the fractal dimension is, the bigger the variation in the cutting force will be. They also established relationships between fractal dimensions of cutting forces and the machining parameters; such relations can be very useful in selecting profiling process parameters for the tested tools and granite material.

The initiation of granite edge finishing research by Bahri et al. (2021) [18] extended the understanding of this domain. Their investigation into edge shaping, utilizing abrasive tools with increasing grit sizes and a concave round shape, illuminated the significance of spindle speed in achieving superior surface finishes, particularly with roughing tools. However, this contradicted the observations by Songmene et al. 2018 [16] where feed rate exhibited less influence on surface finish in plane surface polishing. Additionally, Bahri et al. (2021) [18] underscored the advantages of lubrication, revealing a tenfold reduction in Ra values during dry grinding compared to lubricated processes. Moreover, lubrication enhanced the brightness of edge-polished surfaces (when using the grit 600 tool).

Building upon this knowledge, the recent study by Bahri et al. (2022) [19] delved into the impact of minimum quantity lubrication (MQL) flow on part quality and dust emission during granite edge finishing. The research identified a significant correlation between the MQL flow rate and part quality. However, the study calls for further exploration, urging an investigation into the implications of wet lubrication on part quality across different granite types, as the focus was solely on black granite. Notably, the study also highlights the need for a comprehensive understanding of the interplay between tool geometry, material characteristics, and their combined influence on part quality—a dimension that remains unexplored in the existing body of research.

As we can notice, there have been a very limited number of research activities on natural rock transformation quality and cutting force. On the contrary, there has been a lot of interest in occupational health and safety in granite and marble processing (Ordonez et al. 2007 [20]; Philips and Johnson (2012) [21]; Hall et al. (2022) [22]; Thompson and Qi (2023) [23], etc.). The main reason is that these transformation processes present a high risk to worker health and safety, and high acoustic levels and air pollution indices, as noticed by Mezadre and Bianco (2014) [24]. In recent years, more and more attention has also been paid to artificial stones, which are cheaper and easier to produce than granites, but which present a greater risk due to their high crystalline silica content (Carrieri et al. (2020) [25]; Salamon et al. 2021 [26]). Salamon et al. (2021) [26] found that the risk was higher for workers involved in manual, dry, or wet finishing operations as compared to mechanical operations. The shaping processes for these engineered stones are similar to those for granite and include also edge profiling. Most of the research works on natural and artificial stone finishing are thus focused on dust extraction from the cutting environment, such as Pierce (2019) [27], with less attention to product quality improvement or process optimization. All these processes’ performance should be studied, both for plane finishing and edge profiling.

This article delves into a critical dimension of the edge finishing process, specifically focusing on the abrasive tools involved in refining the edges of granite material products. The investigation encompasses a comprehensive analysis of machining conditions, lubrication methods, shapes and grit sizes of the abrasive tools used, and their effects on the produced edge quality and on cutting forces, under wet and dry machining conditions. By scrutinizing these elements, the aim is to furnish valuable insights that ensure an impeccable surface finish, aligning closely with the exacting requirements of customers. In essence, this research endeavors to unravel the intricacies of tool part quality, recognizing its pivotal role in not only meeting but surpassing customer expectations in the ever-evolving granite transformation and finishing industry.

## 2. Experimental Procedure

Granite edge profile fabrication is a very important step of granite countertop manufacturing (USA Granite Tools, [28]). The success of the process is often evaluated or quickly assessed through the product’s edge quality.

**Figure 1:**
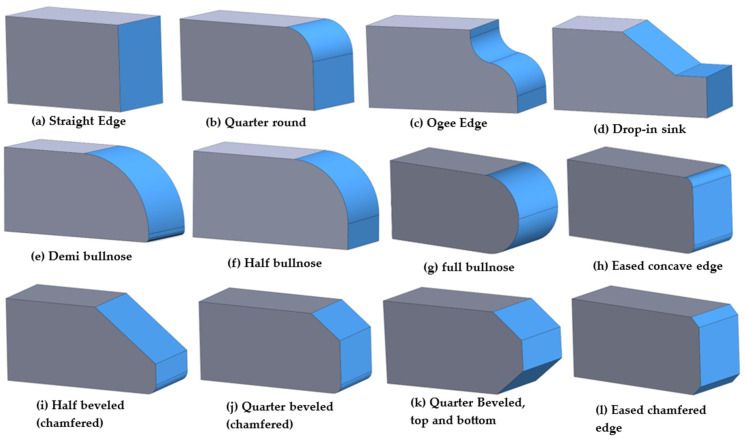
Some popular granite countertop edge profiles (adapted from Alpha profiler shapes [30] and Mogastone 2023 [31]).

There are a wide variety of natural stone and quartz/engineering stone profiling tools on the market for handheld power tools and automatic and CNC machines (USA Granite [28], GranQuartz [29], Alpha Tools [30], etc.). They range from metal bond router bits, profilers with roller guides, and custom electroplated profile wheels to diamond routers. The tool shape used depends on the customers’ desired edge shapes. According to USA Granite Tools [28], the most popular profiles include straight (vertical edge and sharp corners), round, full bullnose profile (having a radius that makes the edge smooth) and half bullnose, ogee profiles (curved edge profiles), waterfall, beveled or chamfered profiles, and eased edge profiles (rounded tops and bottoms); Figure 1 presents some of these most popular granite countertop edge profiles.

**Figure 2:**
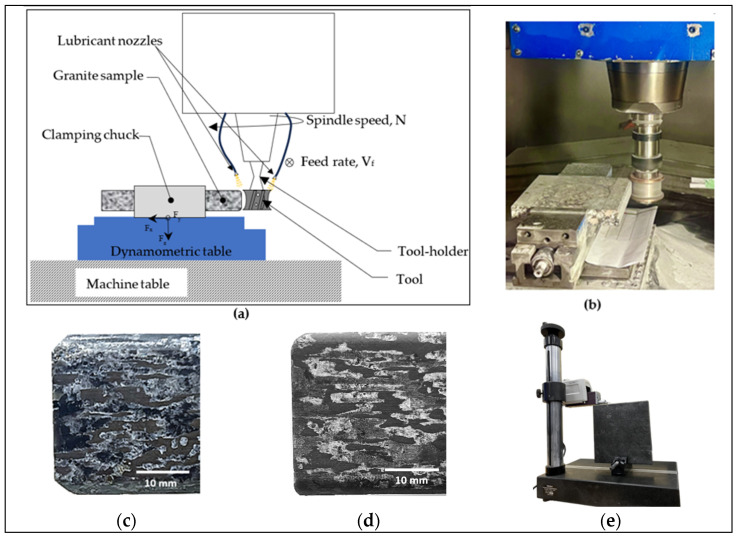
Experimental cutting tests setup: (**a**) testing procedure; (**b**) mounting granite piece on the polishing machine; (**c**) chamfered black granite; (**d**) concave-shaped black granite; and (**e**) roughness measurement devices with Mitutoyo Surftest SJ-410 Mitutoyo America Corporation, Aurora, IL, USA.

In this study, the experiment involved edge finishing consisting of grinding and polishing edges (eased concave edge, Figure 1h and Figure 2d, and eased chamfered edge, Figure 1l and Figure 2c) using varying process parameters for granite products to explore the impact of these parameters, along with the influence of the tool shape, on surface roughness and cutting forces. A comprehensive factorial design of the experiment plan was used. The input factors and their levels are listed in Table 1 while the output responses analyzed are summarized in Table 2. The details of abrasive tools used are given in Table 3. A variety of tools, each featuring distinct grain sizes tailored to the specific edge shapes, were employed during the edge finishing process, as detailed in Table 3. These abrasive tools were purchased from GranQuartz Canada Inc. (Stanstead, QC, Canada).

This design resulted in a total of 144 tests, determined by Equation (1). To enhance the robustness of the analyses, each test was repeated three times, resulting in a cumulative total of 432 tests. These data were used to build the statistical analysis and analysis of variance (ANOVA) presented in this article.

(1) $$\prod {\left(Number\;of\;levels\right)}^{\left(number\;of\;parameters\right)}={2^{4}*3}^{2}=144\;tests$$

The granite workpiece samples utilized in this study were 200 × 200 × 30 mm^3^ of white and black granites donated by A. Lacroix Granit (Saint-Sébastien- -Frontenac, QC, Canada) as part of their contributions to the granite transformation research project. Table 4 summarizes the composition of the workpiece materials tested. The white granite used in our study consisted of 41% quartz, 33% plagioclase, and 23% K-feldspar. The black granite was a Canadian anorthosite with coarse grains, consisting mainly of plagioclase (about 83%). It did not contain quartz. The grain size of black granite (0.2 to 17.0 mm) is greater than that of white granite (0.5 to 7.0 mm), as determined by mineralogical analysis (Bahloul et al., 2019) [32]. The average density of white granite is about 2.7 g/cm^3^, while black granite has a higher average density, of about 3.1 g/cm^3^ (Bahri et al. , 2021) [18].

Figure 2 illustrates the experimental setup. The workpiece was mounted on a dynamometric table and the profiling tools on the spindle of the CNC milling machine (Figure 2a,b). The main objective consisted of studying the generation of chamfered shapes (Figure 2c) and concave shapes (Figure 2d) on granite samples. The edge finishing procedures were executed on a K2X10, 3-axis computer numerical control (CNC) milling machine (Figure 2b) (max spindle speed 28,000 rpm, torque 50 Nm, power 40 kW), manufactured by Huron GRAFFENSTADEN SAS, (Eschau, France). For wet edge finishing, the lubricant was applied using two nozzles (Figure 2a) at a flow rate of 30 L/min and a pressure of 3 bars, while for dry cutting, the lubricant application was switched off.

The cutting forces were measured using the Kistler dynamometric table 9255B (Figure 2a) (Kistler Instrument Corporation, New York, NY, USA), along the three axes (x, y, z), employing a total of 5 sensors for accurate and thorough force acquisition.

The assessment of surface roughness was conducted using a Mitutoyo Surftest SJ-201 profilometer (Mitutoyo America Corporation, Aurora, IL, USA). This instrument features a probing system that scans the surface and generates various roughness parameters such as arithmetic mean deviation of the surface profile (Ra) and total height of the surface profile (Rt). To guarantee accurate measurements, validation was undertaken using the Surftest SJ-410 (Figure 2e) at multiple points throughout the process. Before initiating measurements, the devices underwent calibration using a roughness standard with Ra = 2.95 μm to ensure precision and reliability in the obtained results. The roughness values were measured three times at three different positions on the lateral face processed by each tool shape using a Mitutoyo Surftest SJ-201. The measurements were conducted with a positioning jig designed in the ÉTS workshop. For the chamfer and concave surface roughness, the Surftest SJ-410 (Figure 2e) was used. The latter enables better manipulation of the granite sample during measurement, ensuring full positioning and contact of the detector with the chamfer and concave surfaces.

## 3. Results and Discussion

### 3.1. Effect of Abrasive Grit Size and Workpiece Material on Part Quality

**Figure 3:**
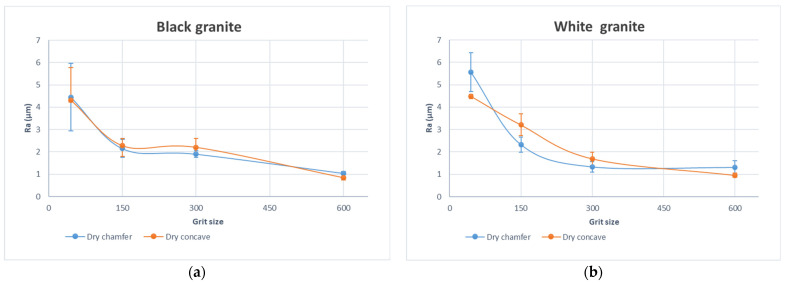
Curves of arithmetic average roughness Ra as a function of grit size for the chamfer and concave shapes with fixed spindle speed (*N* = 2500 rpm) and fixed feed rate (*V_f_* = 1000 mm/min): (**a**) black granite, (**b**) white granite.

**Figure 4:**
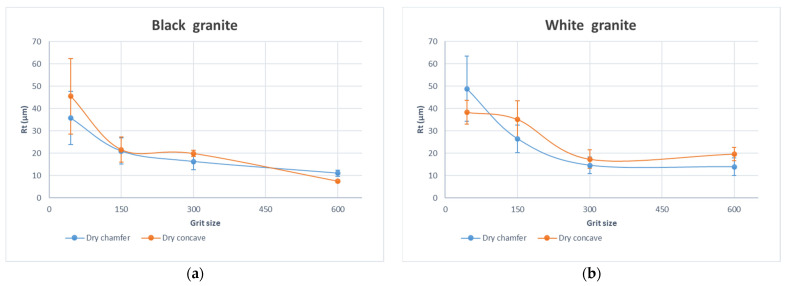
Curves of the total height of the profile Rt as a function of the grit size for the chamfer and concave shapes with fixed spindle speed (*N* = 2500 rpm) and fixed feed rate (*V_f_* = 1000 mm/min): (**a**) black granite, (**b**) white granite.

In the examination of processed granite surfaces, the evolution of Ra values and Rt values, depicted in Figure 3 and Figure 4, respectively, reveal insightful patterns in response to variations in abrasive grit sizes. Emphasizing the significance of grit size as the paramount parameter shaping surface roughness dynamics, the observed trend indicates a substantial decrease in Ra as abrasive grit size increases. This reduction in Ra values reflects an improvement in the average surface roughness, aligning with Ra’s role in specifying acceptable levels of smoothness or roughness across diverse granite industries. Concurrently, the decrease in Rt signifies a reduction in extreme variations, contributing to an enhanced overall surface finish. Notably, the importance of a lower Rt is underscored, as it helps mitigate the risk of surface damage, making it a key consideration for applications where surface integrity is paramount.

The distinctive surface qualities, as measured by Ra and Rt values, observed between black and white granite during edge finishing using various grit sizes (Figure 3 and Figure 4), can be elucidated by their divergent mineralogical and physical characteristics. Black granite, predominantly composed of plagioclase (83.6%) and orthopyroxene (6.85%), exhibits a diverse range of grain sizes spanning from 0.2 to 17 mm (Bahloul et al. 2019) [32]. This wide spectrum of grain sizes, coupled with the lower hardness of plagioclase, facilitates a smoother finish during the edge finishing process. Conversely, white granite, comprising quartz (41.4%), plagioclase, and K-feldspar, presents smaller and more uniform grain sizes ranging from 0.5 to 7 mm (Bahloul et al. 2019) [32]. The higher hardness of quartz (7 on the Mohs scale) in white granite (Mourre 1996) [33], combined with a relatively uniform grain size distribution, may contribute to a textured surface and increased tool wear during the finishing process. Additionally, the significant quartz content in white granite, compared to its proportion in black granite, introduces an abrasive component that can influence tool interactions and impact the final surface quality. In essence, the interplay of mineral composition, grain size distribution, and hardness disparities underscores the nuanced factors influencing the surface finishes of black and white granite during edge finishing.

**Figure 5:**
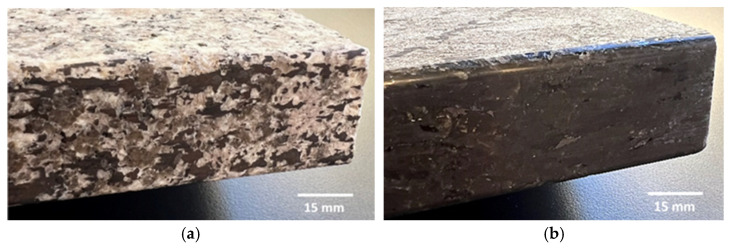
Edge finish of the granite samples (grit 600, *V_f_* = 1000 mm/min, and *N* = 2500 rpm): (**a**) white granite, (**b**) black granite.

The black granite exhibits a marginally superior surface quality compared to the white granite. This distinction is visually evident in Figure 5, where the brighter appearance of the black granite is noticeable in comparison to the white granite.

### 3.2. Effect of Lubrication on Surface Quality

**Figure 6:**
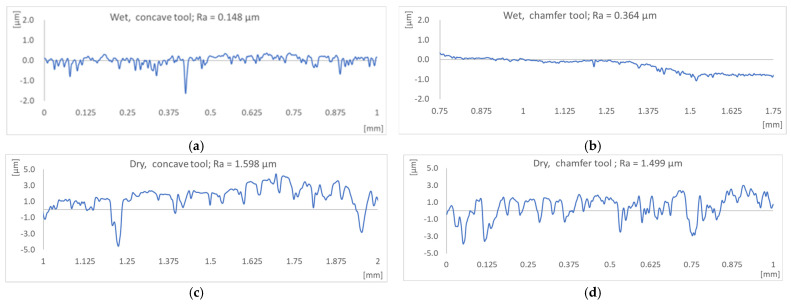
Evolution of the surface profiles and roughness Ra of the black granite, grit 600, *N* = 2500 rpm, *V_f_* = 1000 mm/min: (**a**) wet, concave tool; (**b**) wet, chamfer tool; (**c**) dry, concave tool; (**d**) dry, chamfer tool.

Figure 6 shows the surface roughness profiles obtained when edge finishing black granite in wet and dry conditions using concave and chamfered tools (grit 600). In these graphs, it is evident that working with lubrication consistently yields the lowest roughness values (Ra) for both abrasive tool shapes. This outcome aligns with expectations, as the lubricant effectively reduces friction between the part and the tool while ensuring tool cooling during cutting, thereby contributing to a superior surface finish.

Significantly, there is a notable contrast in the average arithmetic roughness values. Dry edge finishing for the concave shape exhibits up to 10 times greater roughness, while for the chamfer shape, it is 5 times greater compared to lubricated edge finishing. It is evident that lubrication reduces the roughness by two times for the concave shape compared to what it does for the chamfer shape. This observation emphasizes that the chamfer shape inherently generates lower surface quality, a point we will delve into further in the “Effect of tool shape on cutting forces and surface quality” section. Therefore, lubrication becomes imperative when a chamfer shape is requested by a customer. These findings corroborate the work of Bahri et al. (2021) [18], affirming that the use of lubrication consistently produces superior results in terms of both roughness and the overall brightness of the polished edge.

In accordance with Bahri et al. (2022) [19], the significance of lubrication is underscored, as demonstrated by their achievement of a Ra value of 0.502 μm in MQL mode (60 mL/min) and 0.473 μm in flood lubrication for the chamfer shape. However, it is crucial to note that in our study, lubrication in the wet mode resulted in even lower Ra values, reaching 0.148 μm for the concave shape and 0.364 μm for the chamfer shape. This emphasizes the notable impact of lubrication on surface quality in the context of different tool shapes. In general, the arithmetic roughness averages produced in dry conditions using concave or chamfer tools are comparable. In fact, this surface finish is more dependent on tool grit size (which is the same) than on the tool or the desired edge profile shape.

**Figure 7:**
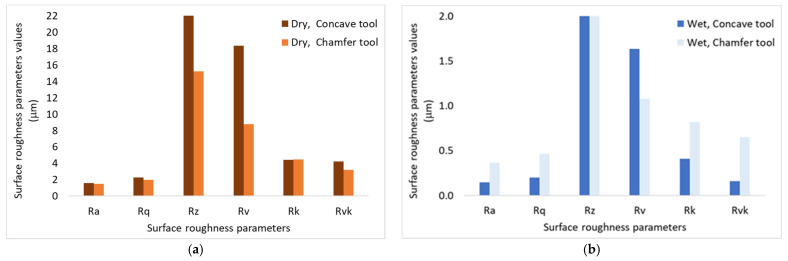
Effects of the cutting fluid on the edge surface roughness parameter values, grit 600, *N* = 2500 rpm, *V_f_* = 1000 mm/min: (**a**) cutting condition: dry; (**b**) cutting condition: wet. Ra: Arithmetic mean deviation. Rq: Root mean square deviation. Rz: Maximum height of the profile. Rv: Maximum profile valley depth. Rk: Core roughness depth. Rvk: Reduced valley height.

In general, using the cutting fluid improved the surface finish of the produced edges (Figure 6 and Figure 7). The flood-cutting fluid helped flush away the generated particles from the cutting zones, thus preventing the particles from rubbing on the surface and contributing to the improvement of tool life as three-body wear could be avoided. The cutting fluid also cools down the cutting area and that contributes to improving the tool performance and avoiding the burning of the workpiece, as has already been pointed out by Bahri et al. (2021, 2022) [18,19]. Figure 7a,b compare some selected roughness parameters for the same profiles presented in Figure 6. They include the arithmetic mean deviation (Ra), the root mean square deviation (Rq), the maximum height of the profile (Rz), the maximum profile valley depth (Rv), the core roughness depth (Rk), and the reduced valley height (Rvk). The core roughness depth (Rk), reduced peak height (Rpk), and reduced valley depth (Rvk) are used to evaluate friction, abrasion, and the capability of the surface to retain liquid. These roughness parameters were better when machined wet using both concave and chamfer tools (Figure 7b).

### 3.3. The Effect of Granite Type and Tool Grit Size on Cutting Forces

**Figure 8:**
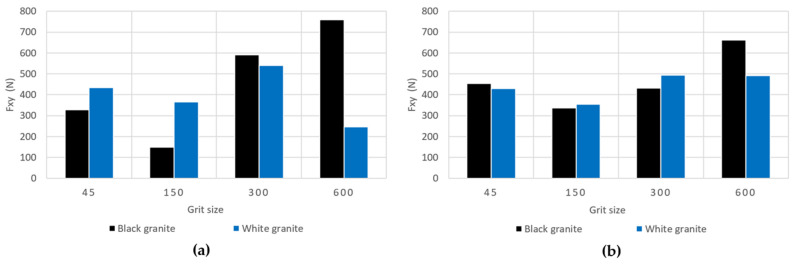
Variation of the combined longitudinal–transversal force F_xy_ as a function of grits for white and black granite with fixed spindle speed (*N* = 2500 rpm) and fixed feed rate (*V_f_* = 1000 mm/min): (**a**) dry concave, (**b**) dry chamfer.

Figure 8a,b illustrate the fluctuation of the combined longitudinal–transversal force, F_xy_, computed using Equation (2).

(2) $$F_{xy}=\sqrt{F_{x}^{2}+F_{y}^{2}}$$

From these charts (Figure 8a,b), it is evident that the cutting forces during the edge finishing of black granite are marginally higher than those encountered when edge finishing white granite, applicable to both dry concave and dry chamfer conditions. This observation aligns with the findings of Preston (1927) [34], who established that black granite demands more energy for polishing compared to white granite in dry concave settings. This discrepancy is attributed to the higher coefficient of friction and density of black granite, surpassing those of white granite. However, further investigation is warranted to delve deeper into this phenomenon.

**Figure 9:**
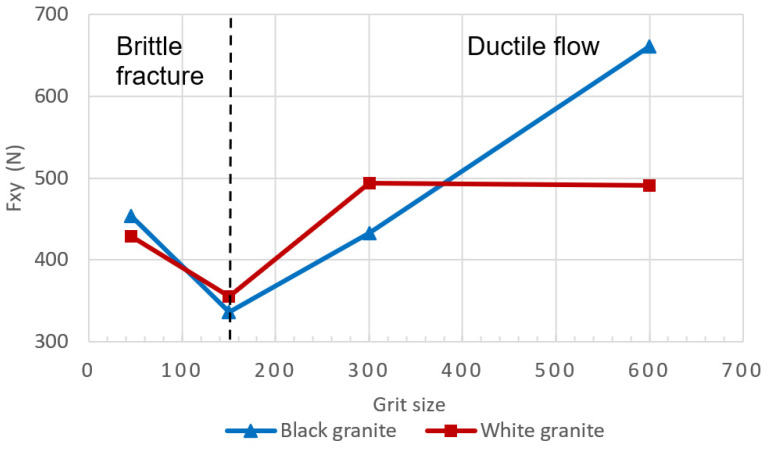
Variation of the combined longitudinal–transversal force F_xy_ as a function of grits for white and black granite for dry chamfer (*N* = 2500 rpm; *V_f_* = 1000 mm/min).

We observe a noteworthy pattern in the combined longitudinal–transversal forces F_xy_ (Figure 9), where a decrease is evident between 45 and 150 grit size, followed by an increase between 300 and 600 grit size. This shift can be elucidated by considering the initial stages of the procedure, where contact predominantly occurs between coarse grits and the rugged surface of the granite. In this phase, the material removal mechanism is characterized by a brittle fracture (Xu et al. (2003)) [7], signifying that larger abrasive grits effectively remove substantial material, enlarging the contact surface and consequently reducing contact pressure. The heightened contact surface between the coarse surface and large abrasive grits leads to an increase in the friction coefficient, thereby reducing the forces, as established by Saidi et al. (2015) [6]. Contrastingly, in the final stages, contact is established between finer grits and the polished surface of the granite. Here, the material removal mechanism shifts to a ductile flow (Xu et al. (2003)) [7], resulting in a diminished quantity of material being removed. The contact surface is now contingent on the size of the abrasive grit, and as smaller grits are employed, the surface becomes smoother with a reduced contact area. This, in turn, leads to heightened contact pressure and a consequent decrease in the friction coefficient.

### 3.4. Effect of Tool Shape on Cutting Forces and Surface Quality

**Figure 10:**
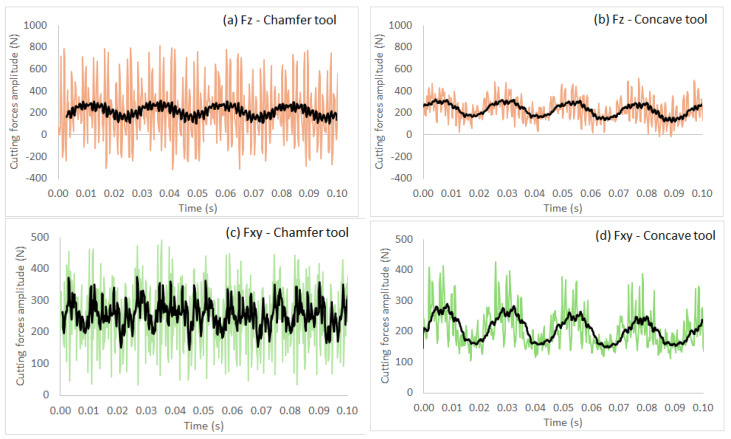
Variation over time of forces (tool grit: 600; black granite; *N* = 2500 rpm; *V_f_* = 1500 mm/min; dry conditions): (**a**) Fz—chamfer tool; (**b**) Fz—concave tool; (**c**) Fxy—chamfer tool; and (**d**) Fxy—concave tool.

Figure 10a–d compares the cutting force profiles obtained when using a grit 600 chamfer tool and a grit 600 concave tool to edge finish black granite at a spindle speed of 2500 rpm and a feed rate of 1500 mm/min in dry conditions. The black lines in these graphs are the moving average tendencies (period of 20 data). These tendencies oscillate around 200 N for the axial force (F_z_) and at about 250 N for the plane force (F_xy_). The regular patterns in these graphs can be explained by the slots on the profiling tools (Table 3). The maximum cutting forces (F_z_ and F_xy_) recorded were relatively higher for the chamfer profiling tool as compared to the concave tool used in the same cutting conditions. This is the result of the contact points between the chamfered tool and the workpiece.

**Figure 11:**
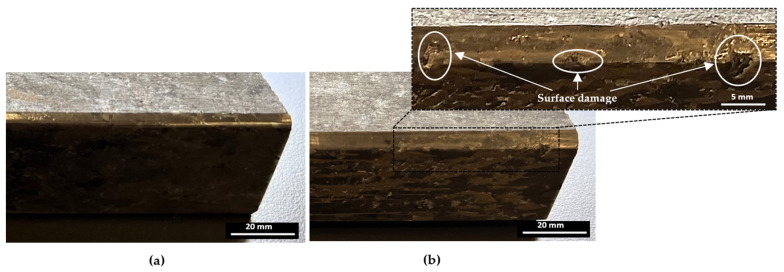
Tool shape effect on surface quality (wet lubrication; *N* = 2500 rpm; *V_f_* = 1500 mm/min): (**a**) concave tool shape, (**b**) chamfer tool shape.

Edge finishing with a chamfer shape introduces fluctuations in cutting efforts, resulting in a lack of stability. This instability contributes to excessive material removal, elevating the risk of chipping and surface damage. This effect is discernible in Figure 11, where the granite edge finished with the chamfered abrasive tool exhibits pronounced material removal and surface damage. These damages can also be the result of poor workpiece material quality at the locations where the damages were observed. Conversely, the granite edge finished with a concave shape tool showcases a superior, more refined surface finish, emphasizing the impact of tool geometry on the overall quality of the edges finished.

**Figure 12:**
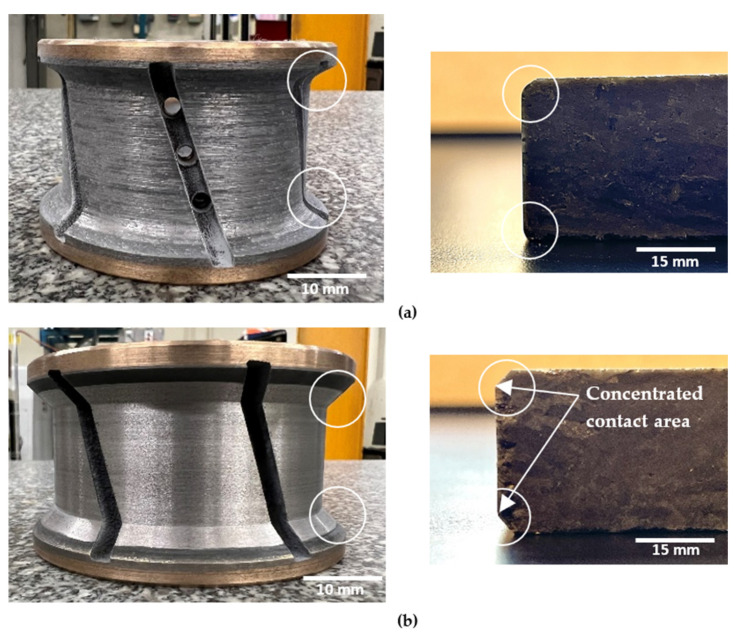
Illustration of the characteristics and shapes produced with each tool: (**a**) concave shape, (**b**) chamfer shape.

The concave shape, distinguished by its gracefully curved surface (Figure 12a), excels in imparting a smoothing effect during the edge finishing process. It facilitates the even distribution of contact pressure across the granite surface, in stark contrast to the more localized contact points inherent to the chamfer shape tool. This characteristic of the concave shape tool serves to mitigate the risk of surface damage, thereby elevating the overall finish. Furthermore, the curvature of the concave shape enables precise control over material removal, preventing undue abrasion and ensuring a consistent removal of material. This controlled process contributes significantly to achieving a smoother surface.

Conversely, the chamfer shape tool, as illustrated in Figure 12b, tends to have a more concentrated contact area with the granite workpiece surface than its concave counterpart. This concentrated contact elevates the pressure at specific points, potentially resulting in increased forces during the edge finishing operation. Consequently, surface damage is predominantly located in this concentrated contact area, as illustrated in Figure 11b. Additionally, the chamfer shape tool may experience less stable tool engagement compared to the concave shape tool, introducing variability in the edge finishing process and leading to fluctuations in cutting forces.

### 3.5. Analysis of Variance for the Surface Finish

The primary goal of implementing the analysis of variance (ANOVA) was to assess the significance of parameters influencing the roughness parameter (Ra) and the cutting forces F_xy_ and F_z_ in our study. ANOVA analysis was conducted at a 10% significance level and a 90% confidence level to thoroughly examine the impact of various factors.

Given our strong recommendation for the use of wet lubrication mode, the design of experiments (DOE) was meticulously tailored to assess the influence of spindle speed and feed rate across different tool shapes and grit size configurations. The objective was to formulate empirical models for each specific configuration, as meticulously detailed in Table 5. The exploration of cutting parameters’ impacts inherently hinges on the characteristics of the employed tool, encompassing both its shape and grit size. This dependency arises from the essential progression through tools with ascending grit sizes during edge finishing, and specific tool shapes may be explicitly required for the task at hand.

In Table 6a, it is evident that among the parameters examined, only the spindle speed (N) demonstrated a noteworthy impact on the roughness (Ra), as indicated by a *p*-value below 0.05, corresponding to a 95% confidence interval. This influence was characterized by its quadratic form (N^2^), yielding an F-ratio of 0.023, followed by its linear form (N) with an F-ratio of 0.027. Table 6b indicates that no significant effects (at 95% confidence interval) among the studied parameters are evident. The ANOVA for surface finish was done mainly for the grit 600 (finishing phase).

Surface quality is of paramount importance in edge finishing from a manufacturing perspective, with specific emphasis on the finishing tool’s ability to deliver superior results. Consequently, our statistical investigation concentrated solely on the cutting parameters employed during the finishing phase, specifically using a grit size of 600 and a wet lubrication mode (Table 6).

The outcomes of the ANOVA analysis reveal that, although the effects on the Ra response were statistically significant for both spindle speed and feed rate, the order of significance varied between the two. Detailed in Table 6a, the findings demonstrate that the spindle speed played a predominant role, contributing 58.81% when utilizing the chamfer tool. In contrast, the feed rate exhibited minor impacts, contributing only 23.72%.

In Table 6a, notable significance was observed during edge finishing with the chamfer tool, particularly in the quadratic form of the feed rate (*V_f_*^2^). Following this, the linear form of spindle speed (*N*), the linear form of the feed rate (*V_f_*), and the quadratic form of spindle speed (*N*^2^) exhibited significance in descending order. These influential parameters were incorporated into a linear regression model (Equation (3)). The resulting model demonstrated a strong correlation with an R^2^ value of 93%.

(3) $${{R_{a}\left(Grit\;600,\;Chamfer\right)=10^{-3}\left(1.98N-5.16V_{f}\right)-10}^{-7}(3.76N}^{2}{-3.41V}_{f}^{2})$$

**Figure 13:**
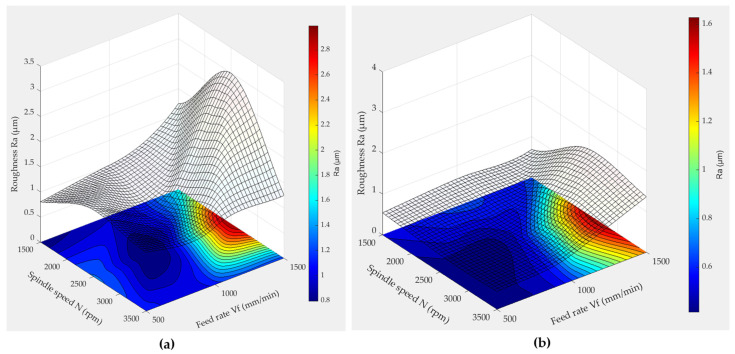
3D response surface plots of Ra when using a concave shape tool (grit 600): (**a**) dry lubrication mode, (**b**) wet lubrication mode.

**Figure 14:**
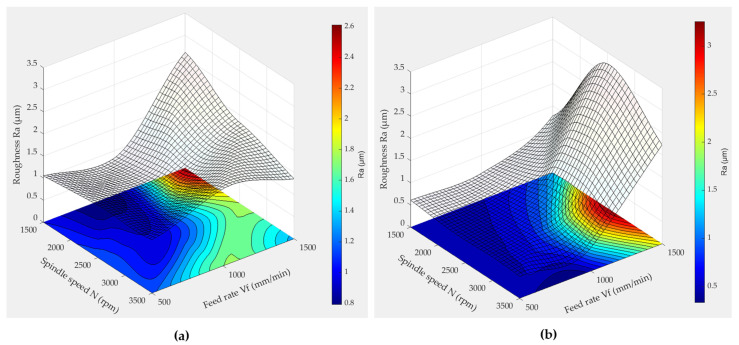
3D response surface plots of Ra when using a chamfer shape tool (grit 600): (**a**) dry lubrication mode, (**b**) wet lubrication mode.

The response surface methodology was employed to analyze the evolution of the roughness parameter Ra during the finishing phase, utilizing both chamfer and concave shape tools. Figure 13 and Figure 14 showcase the 3D plots generated by this method, illustrating the variation in roughness Ra as a function of feed speed (*V_f_*) and spindle speed (*N*) under conditions of 600 grit size and wet lubrication.

In these 3D graphs (Figure 13 and Figure 14), regions with minimal roughness Ra are depicted in blue, whereas larger roughness values are represented by the color red. It is evident that low roughness values are predominantly achieved when using lubrication with low feed rates and high spindle speeds when employing the chamfer shape tool. When utilizing the concave shape tool, consistently low roughness values Ra are attained within a spindle speed range of 2250 rpm to 3500 rpm, especially when the feed rate is minimal.

The influence of lubrication is evident in the contrasting Figure 13a,b as well as Figure 14a,b, showcasing a noticeable reduction in the roughness Ra value, essentially halving it and resulting in an enhanced surface quality. The elevated Ra value that is observed in Figure 14b, particularly when employing a spindle speed of 2500 and a feed rate of 1500, can be attributed to the inherent characteristics of the chamfer shape. It appears that, even with the application of lubrication underscoring the significance of lubrication in diminishing roughness and improving overall surface quality, the chamfer shape may induce surface damage.

Analysis of Figure 14 indicates that a higher feed rate corresponds to an elevated roughness value (Ra). This relationship is anticipated, as elevated feed rates imply a faster advancement of the cutting tool along the workpiece. The swifter movement leads to a higher material removal rate, and when the material is removed too rapidly, the cutting tool may struggle to effectively engage with the surface. This inefficiency in engagement can result in uneven cutting and contribute to the observed rise in roughness. In their study on the surface roughness of granite machined by abrasive waterjet, Aydin et al. (2011) [35] developed an explanation for the observed increase in roughness associated with higher travel speeds. They attributed this phenomenon to a reduced number of particles passing through a unit area as the waterjet’s speed increased. Consequently, the accelerated movement resulted in fewer impacts and cutting edges available per unit area, leading to the formation of rougher surfaces. Moreover, the study by Bahri et al. (2021) [18], focusing on edge finishing, similarly noted variations between edge finishing and surface polishing results, contrasting with the findings of Songmene et al. (2018) [16] on plane polishing, whose work indicated that higher feed rates contribute to improved surface conditions. It is important to note the potential influence of process variations, particularly in comparing surface polishing to edge finishing. This discrepancy warrants further investigation.

This observation underscores the significance of optimizing machining parameters. Specifically, a synergistic combination of low feed rates and high spindle speeds proves beneficial, allowing the tool to effectively polish the edge surface under favorable conditions that mitigate wear and prevent excessive heating. This optimization is crucial, as unfavorable tool conditions are indicative of suboptimal surface finishes across various machining and polishing processes.

### 3.6. Analysis of Variance for Cutting Forces

Similarly, the analysis of variance was also carried out on the cutting forces data (F_xy_ and F_z_) for all tested tools, focusing on grit 150 and grit 600. The obtained ANOVA is presented in Table 7. The presented ANOVA results (Table 7) offer valuable insights into the intricate relationship between cutting conditions and forces in CNC edge grinding processes.

In general, when machining with a rougher tool (grit 150), the cutting forces are governed by the tool rotational speed and the feed rate, while for the finishing process, it is the tool rotational speed that is the most influential factor. This can be explained by the material removal modes that are different in these two process phases.

Focusing on the chamfer-shaped tool with a 150 grit size, a detailed examination of Table 7a reveals that all design of experiments (DOE) parameters significantly influence the combined longitudinal–transversal force, F_xy_. The most significant impact is attributed to the spindle speed (*N*), evident in the highest F-ratio value, followed by the feed rate (*V_f_*) and their interaction (*N* × *V_f_*). Utilizing these significant parameters and their interaction, we formulated an empirical model encapsulated in Equation (4), effectively representing 93% of the F_xy_ data with an adjusted R^2^ of 89%. Similarly, the influence of all tested parameters on F_z_ is significant (Table 7b), mirroring the hierarchy observed for F_xy_. The empirical model, expressed in Equation (5), faithfully represents 96% of the F_z_ data, with an adjusted R^2^ of 94%.

When employing the chamfer tool shape with a 600 grit size, the combined longitudinal–transversal force, F_xy_, demonstrates substantial sensitivity to both spindle speed (N) and the interaction between spindle speed and feed rate (*N* × *V_f_*). These crucial parameters were integrated into a robust linear regression model (Equation (6)) with an impressive correlation coefficient (R^2^ = 98%) and an adjusted R^2^ of 97%. Conversely, for F_z_, only the spindle speed (N) exhibited a significant effect, resulting in the formulation of an empirical model as depicted in Equation (7), showcasing an R^2^ of 94% and an adjusted R^2^ of 93%.

In the application of the concave tool shape with a 150 grit size, the combined longitudinal–transversal force, F_xy_, demonstrates notable sensitivity to both spindle speed (*N*) and feed rate (*V_f_*). These pivotal parameters were incorporated into a robust linear regression model (Equation (8)) with a correlation coefficient (R^2^ = 87%) and an adjusted R^2^ of 83%. Additionally, forces along the *Z*-axis (F_z_) exhibit a strong correlation with all DOE parameters, following the same hierarchy observed for F_xy_ and F_z_ when using the chamfer-shaped tool with a 150 grit size. The model representing this correlation for the concave tool is given by Equation (9), showcasing a high R^2^ of 96% and an adjusted R^2^ of 94%.

In the context of the 600 grit size, only the spindle speed (*N*) exhibited a substantial impact on F_xy_, as indicated by Equation (10), which boasts an R^2^ of 94% and an adjusted R^2^ of 93%. Conversely, in the case of F_z_, both spindle speed (*N*) and feed rate (*V_f_*) contributed significantly, culminating in an empirical model outlined in Equation (11). This model is distinguished by an impressive R^2^ of 96% and an adjusted R^2^ of 95%.

The obtained empirical equations for cutting forces as a function of the cutting parameters (spindle speed *N* and feed rate *V_f_*) and according to ANOVA results (using only statistically significant terms) are as follows:

(a) For grit 150 chamfer tools used in wet condition

(4) $$F_{xy}\left(Grit\;150,\;Chamfer\right)=9.47\cdot 10^{-2}N+1.64\cdot 10^{-1}V_{f}-7.5\cdot 10^{-5}N\cdot V_{f};R^{2}=93\%$$

(5) $$F_{z}\left(Grit\;150,\;Chamfer\right)=1.13\cdot 10^{-1}N+2.25\cdot 10^{-1}V_{f}-9.6\cdot 10^{-5}N\cdot V_{f};R^{2}=96\%$$

(b) For grit 600 chamfer tools used in wet condition

(6) $$F_{xy}\left(Grit\;600,\;Chamfer\right)=0.2\cdot N+1.05\cdot 10^{-4}N\cdot V_{f};R^{2}=98\%$$

(7) $$F_{z}\left(Grit\;600,\;Chamfer\right)=3.41\cdot 10^{-1}N;R^{2}=94\%$$

(c) For grit 150 concave tools used in wet condition

(8) $$F_{xy}\left(Grit\;150,\;Concave\right)=6.74\cdot 10^{-2}N+10^{-1}V_{f};\;R^{2}=87\%$$

(9) $$F_{z}\left(Grit\;150,\;Concave\right)=1.21\cdot 10^{-1}N+2.45\cdot 10^{-1}V_{f}-9.8\cdot 10^{-5}N\cdot V_{f};\;\;\;R^{2}=95\%$$

(d) For grit 600 concave tools used in wet condition

(10) $$F_{xy}\left(Grit\;600,\;Concave\right)=3.01\cdot 10^{-1}N;R^{2}=93\%$$

(11) $$F_{z}\left(Grit\;600,\;Concave\right)=1.1\cdot 10^{-1}N+4.5\cdot 10^{-1}V_{f};R^{2}=96\%$$

**Figure 15:**
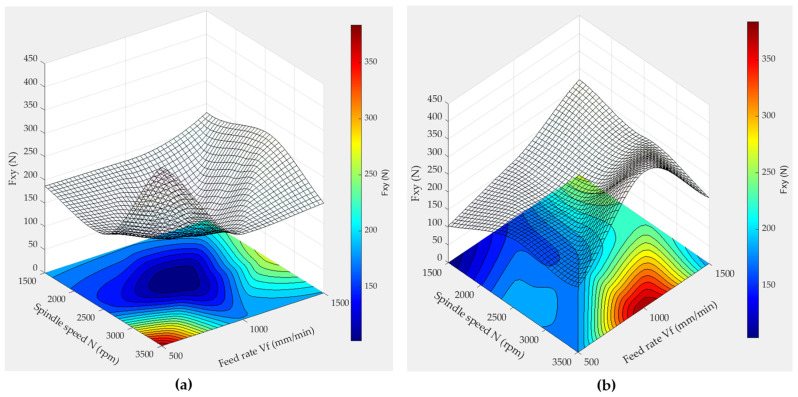

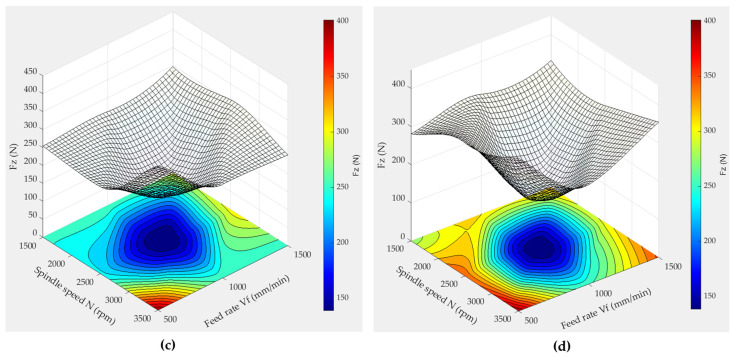
3D response surface plots of F_xy_ and F_z_ when using wet lubrication mode and 150 grit size: (**a**) F_xy_ for chamfer shape tool, (**b**) F_xy_ for concave shape tool, (**c**) F_z_ for chamfer shape tool, (**d**) F_z_ for concave shape tool.

**Figure 16:**
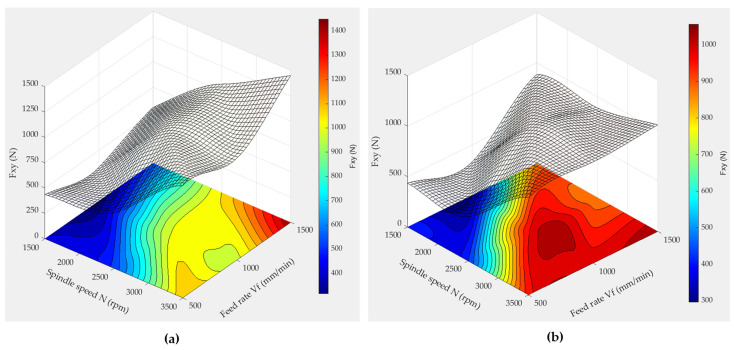

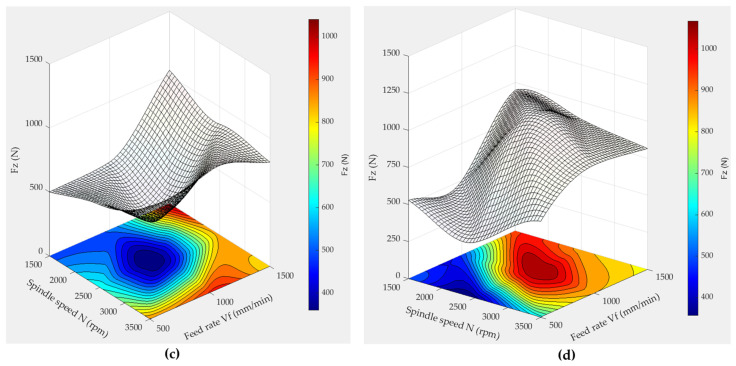
3D response surface plots of F_xy_ and F_z_ when using wet lubrication mode and 600 grit size: (**a**) F_xy_ for chamfer shape tool, (**b**) F_xy_ for concave shape tool, (**c**) F_z_ for chamfer shape tool, (**d**) F_z_ for concave shape tool.

Figure 15 presents the surface responses of cutting forces when edge finishing with grit 150 tools in wet conditions, according to Equations (4), (5), (8), and (9), but considering quadratic terms. Similarly, Figure 16 presents the surface responses of cutting forces when edge finishing with grit 600 tools in wet conditions, according to Equations (6), (7), (10), and (11) and including quadratic terms.

The observed trends in forces (F_xy_ and F_z_) during granite edge grinding, illustrated in the 3D surface plots, unveil distinct patterns associated with varying spindle speeds, feed rates, and grit sizes. In Figure 15, for the 150 grit size, the combination of a spindle speed of 2500 rpm and a feed rate of 1000 mm/min is correlated with the lowest F_xy_ and F_z_ forces. This phenomenon is attributed to the delicate balance between material removal and frictional forces at the tool–granite interface. The higher spindle speed at 2500 rpm likely enhances efficient material removal, while the moderate feed rate of 1000 mm/min ensures controlled contact with the workpiece, minimizing forces. This combination may lead to a more stable cutting process, reducing vibrations and consequently lowering the cutting forces.

Conversely, for the 600 grit size (Figure 16), the optimal conditions shift to a spindle speed of 1500 rpm and a feed rate of 500 mm/min, showcasing the lowest F_xy_ and F_z_ forces. This change is attributed to the finer grit size, influencing the abrasive action on the granite surface. The lower spindle speed and feed rate may be more suitable for the finer abrasive particles, resulting in reduced cutting forces. Thus, the finer grit size is associated with a smoother cutting action, while the lower spindle speed and feed rate contribute to precise material removal, leading to minimized forces.

It is noteworthy that the 600 grit size tends to generate approximately twice the forces F_xy_ and F_z_ compared to the 150 grit size. This could be explained by the 600 grit size being characterized by a ductile flow mechanism, representing the finishing phase, while the 150 grit size is characterized by a brittle fracture mechanism, representing the roughing phase, as discussed in the effect of granite type on the cutting forces section_._

## 4. Conclusions

This experimental investigation illuminates critical aspects of granite edge finishing, highlighting the intricate relationship between granite type, tool geometry, cutting conditions, and part quality. This study underscores the significant impact of abrasive grit size, spindle speed, feed rate, lubrication mode, and tool geometry on surface roughness, challenging established norms in the literature. Drawing from the experiments performed in this study, the subsequent conclusions can be derived:

1. The best surface quality was achieved at a spindle speed of 2500 rpm, while higher feed rates consistently resulted in increased arithmetic roughness values (Ra).
2. Cutting forces obtained during edge finishing of black granite were marginally higher than those obtained for white granite, especially in dry concave and dry chamfer conditions. This observation aligned with historical findings, indicating that black granite demands more energy for edge finishing due to higher friction coefficients and density.
3. Lubrication emerges as a key factor in achieving good surface quality, with wet processes consistently outperforming dry methods, yielding the lowest roughness values for both chamfer and concave shapes. This study validated the expected outcome, as lubrication effectively reduced friction between the part and the tool and ensured tool cooling during cutting.
4. The concave tool shape proves superior to the chamfer tool shape in achieving a good surface finish. The concave shape’s ability to distribute contact pressure evenly contributes to a controlled material removal process, minimizing the risk of surface damage. On the contrary, the chamfer shape’s concentrated contact area introduces variability and instability, potentially leading to surface imperfections.

It is crucial to emphasize that the utilization of a concave shape tool ensures superior surface quality. However, in instances where a chamfer shape is specifically requested by the customer, we highly advocate for the integration of lubrication as a pivotal element. Lubrication plays a key role in reducing friction between the workpiece and the tool, especially in the concentrated contact area. This, consequently, leads to a notable decrease in Ra value and an enhancement in overall surface quality. Moreover, while adjustments to cutting parameters such as spindle speed and feed rate contribute to a reduction in surface roughness, their impact is relatively limited compared to the significant influence of lubrication.

In light of these findings, our recommendations to the constructor involve a strategic emphasis on improving the design of the chamfer shape tool. Specifically, we propose exploring solutions to incorporate a connection radius for the chamfer-shaped tool while preserving the intended appearance of the chamfer shape. This proposed modification aims to minimize chipping in the chamfer shape, further enhancing the overall performance and quality of the machining process.

The outcomes of this research offer valuable insights for the granite processing industry, providing guidance to optimize edge finishing processes concerning the impact of lubrication and specified tool shapes on surface quality. Additionally, this study dissects the effects of individual tool shapes, proposing solutions to achieve desired shapes and ensure excellent surface quality to meet evolving customer expectations. The literature currently lacks comprehensive coverage of these aspects, underscoring the need for further exploration. Specifically, understanding the implications of wet and dry lubrication on part quality across different stone materials during edge finishing requires more attention. This study initiates a call for future research to delve deeper into the intricate dynamics of tool–part interactions, aiming to address the evolving challenges in the granite industry.

## Figures and Tables

**Table 1:** Input factors and their levels.

Factors		Levels	
	1	2	3
Granite type	Black	White	-
Tool shape	Chamfer 3 mm × 45°	Concave 3 mm radius	-
Lubrication mode	Wet	Dry	-
Tool grit size	150	600	-
Spindle speed N (rpm)	1500	2500	3500
$\mathrm{Feed}\;\mathrm{rate}\;V_{f}$ (mm/min)	500	1000	1500

**Table 2:** Output responses studied.

Responses		Description
Roughness	Ra (μm)	Arithmetic mean deviation of the surface profile
	Rt (μm)	Total height of the surface profile
Forces	F_x_ (N)	Cutting forces follow the x-axis
	F_y_ (N)	Cutting forces follow the y-axis
	F_z_ (N)	Cutting forces follow the z-axis

**Table 3:** Details of used abrasive tools.

Grit Size	Chamfer Tools and Dimensions (mm)		Concave Tools and Dimensions (mm)	
150	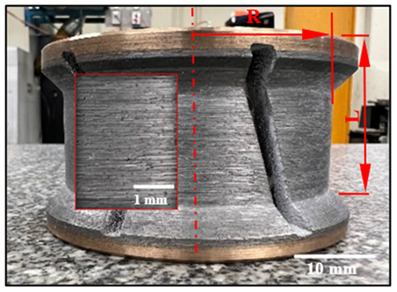	L = 37.4 R = 34.7	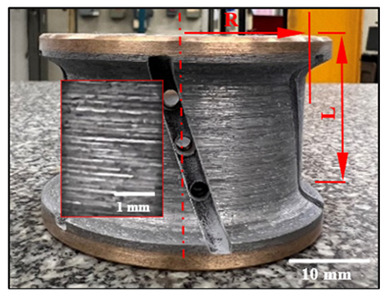	L = 39.0 R = 30.0
600	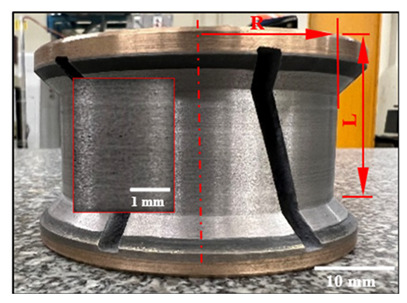	L = 37.6 R = 34.3	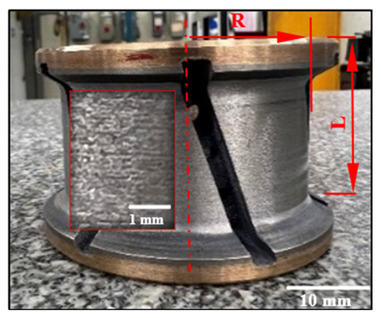	L = 37.5 R = 30.0

**Table 4:** The composition of workpiece materials was tested.

Minerals	Materials and Composition	
	Black Granite (Canadian Anorthosite)	White Granite
Quartz	0%	41.4%
Plagioclase	83.6%	32.4%
K-feldspar	-	23%
Orthopyroxene	6.85%	-
Biotite	2.83%	1.14%
Oxides	5.2%	2 %

**Table 5:** Design of experiments (DOE) for chamfer and concave tool shapes using wet lubrication mode.

Edge Shape	Grit Size	Test Number	Spindle Speed *N* (rpm)	Feed Rate *V_f_* (mm/min)
Concave	150	1	3500	1500
		2	1500	1000
		3	3500	500
		4	2500	500
		5	1500	500
		6	2500	1000
		7	3500	1000
		8	1500	1500
		9	2500	1500
	600	1	3500	500
		2	2500	500
		3	2500	1500
		4	1500	1000
		5	3500	1000
		6	2500	1000
		7	1500	500
		8	1500	1500
		9	3500	1500
Chamfer	150	1	1500	500
		2	2500	500
		3	1500	1000
		4	3500	1500
		5	2500	1000
		6	3500	1000
		7	3500	500
		8	2500	1500
		9	1500	1500
	600	1	2500	1000
		2	3500	500
		3	1500	500
		4	2500	1500
		5	1500	1500
		6	1500	1000
		7	3500	1000
		8	3500	1500
		9	2500	1000

**Table 6:** ANOVA tables for roughness Ra with 600 grit size and wet lubrication mode based on tool shape. (bold P-values indicate significant factors and interactions).

**(a) ANOVA of Ra for Wet Chamfer/Grit 600**						
**Source**	**DF**	**SS**	**MS**	**F-Ratio**	***p*-Value**	**Contribution**
*N*	1	11.44	1.35	5.65	**0.076**	58.81%
*V_f_*	1	4.61	1.31	5.50	**0.079**	23.72%
*N* ^2^	1	0.12	1.29	5.42	**0.080**	0.62%
*V_f_* ^2^	1	1.92	1.39	5.84	**0.073**	9.86%
*N* × *V_f_*	1	0.41	0.41	1.71	0.261	2.09%
Error	4	0.95	0.24			4.89%
Total	9	19.46				100.00%
**(b) ANOVA of Ra for Wet Concave/Grit 600**						
*N*	1	5.07	0.17	2.57	0.184	75.60%
*V_f_*	1	1.07	0.09	1.41	0.301	16.00%
*N* ^2^	1	0.01	0.18	2.72	0.174	0.12%
*V_f_* ^2^	1	0.16	0.09	1.33	0.313	2.40%
*N* × *V_f_*	1	0.12	0.12	1.85	0.245	1.86%
Error	4	0.27	0.07			4.02%
Total	9	6.71				100.00%

**Table 7:** ANOVA tables for forces F_xy_ and F_z_ based on tool shape and grit size. (bold P-values indicate significant factors and interactions).

**(a) ANOVA of F_xy_ for Wet Chamfer/Grit 150**					
**Source**	**DF**	**SS**	**MS**	**F-Ratio**	***p*-Value**
*N*	1	385,935	79,788	14.15	**0.009**
*V_f_*	1	4765	27,275	4.84	**0.070**
*N* × *V_f_*	1	25,160	25,160	4.46	**0.079**
Error	6	33,839	5640		
Total	9	449,699			
**(b) ANOVA of F_z_ for Wet Chamfer/Grit 150**					
*N*	1	605,417	113,247	25.50	**0.002**
*V_f_*	1	12,851	51,270	11.55	**0.015**
*N* × *V_f_*	1	40,519	40,519	9.12	**0.023**
Error	6	26,645	4441		
Total	9	685,432			
**(c) ANOVA of F_xy_ for Wet Chamfer/Grit 600**					
**Source**	**DF**	**SS**	**MS**	**F-Ratio**	***p*-Value**
*N*	1	7,216,895	388,316	17.98	**0.005**
*V_f_*	1	233,491	6731	0.31	0.597
*N* × *V_f_*	1	1599	110,660	5.12	**0.064**
Error	6	226,029	21,594		
Total	9	7,678,014			
**(d) ANOVA of F_z_ for Wet Chamfer/Grit 600**					
*N*	1	7,216,895	425,977	11.31	**0.015**
*V_f_*	1	233,491	81,609	2.17	0.191
*N* × *V_f_*	1	1599	1599	0.04	0.844
Error	6	226,029	37,672		
Total	9	7,678,014			
**(e) ANOVA of F_xy_ for Wet Concave/Grit 150**					
**Source**	**DF**	**SS**	**MS**	**F-Ratio**	***p*-Value**
*N*	1	664,449	92,576	7.30	**0.035**
*V_f_*	1	23,389	52,496	4.14	**0.088**
*N* × *V_f_*	1	29,125	29,125	2.30	0.180
Error	6	76,044	12,674		
Total	9	793,006			
**(f) ANOVA of F_z_ for Wet Concave/Grit 150**					
*N*	1	773,135	129,932	17.84	**0.006**
*V_f_*	1	19,075	60,711	8.34	**0.028**
*N* × *V_f_*	1	42,558	42,558	5.84	**0.052**
Error	6	43,690	7282		
Total	9	878,459			
**(j) ANOVA of F_xy_ for Wet Concave/Grit 600**					
Source	DF	SS	MS	F-Ratio	*p*-Value
*N*	1	5,734,501	362,199	11.00	**0.016**
*V_f_*	1	190,369	83,080	2.52	0.163
*N* × *V_f_*	1	15	15	0.00	0.983
Error	6	197,560	32,927		
Total	9	6,122,446			
**(h) ANOVA of F_z_ for Wet Concave/Grit 600**					
*N*	1	4,563,710	201,607	6.56	**0.043**
*V_f_*	1	474,233	366,295	11.92	**0.014**
*N* × *V_f_*	1	41,949	41,949	1.36	0.287
Error	6	184,430	30,738		
Total	9	5,264,322			

## Data Availability

Data are available upon request, subject to institution restrictions.
